# Remission with or without comorbid substance use disorders in early psychosis: long-term outcome in integrated care (ACCESS III study)

**DOI:** 10.3389/fpsyg.2023.1237718

**Published:** 2023-12-21

**Authors:** Friederike Rühl, Martin Lambert, Anja Rohenkohl, Vivien Kraft, Anne Daubmann, Brooke C. Schneider, Daniel Luedecke, Anne Karow, Jürgen Gallinat, Gregor Leicht, Daniel Schöttle

**Affiliations:** ^1^Psychosis Centre, Department of Psychiatry and Psychotherapy, Centre for Psychosocial Medicine, University Medical Center Hamburg-Eppendorf, Hamburg, Germany; ^2^Department of Medical Biometry and Epidemiology, University Medical Center Hamburg-Eppendorf, Hamburg, Germany

**Keywords:** first episode psychosis, substance use, integrated care, assertive community treatment, remission, recovery

## Abstract

**Introduction:**

Schizophrenia-Spectrum-Disorders are associated with poor long-term outcome as well as disability and often severely affect the lives of patients and their families often from symptom onset. Up to 70% of first episode psychosis (FEP) patients suffer from comorbid substance use disorders (SUD). We aimed at studying the course of illness in FEP patients within evidence-based care, with and without comorbid SUD, to examine how decreased, remitted or persistent substance use impacted rates of a combined symptomatic and functional long-term recovery compared with patients without SUD.

**Methods:**

ACCESS III is an integrated care model for FEP or patients in the early phase of non-affective and affective psychotic disorders. Treatment trajectories of patients, who had been in ACCESS care for 1 year, with and without SUD were compared with regard to the course of illness and quality of life using Mixed Model Repeated Measures (MMRM) and recovery rates were compared using binary logistic regression. Change in substance use was coded as either persistent, decreased/remitted or no use.

**Results:**

ACCESS III was a prospective 1-year study (*N* = 120) in patients aged 12–29 years. Of these, 74 (61.6%) had a comorbid SUD at admission. There were no group differences regarding the course of illness between patients with or without comorbid SUD or between patients with a substance abuse or substance dependence. The only outcome parameter that was affected by SUD was quality of life, with larger improvement found in the group without substance use (*p* = 0.05) compared to persistent and remitted users. Using LOCF, 44 patients (48.9%) fulfilled recovery criteria at the endpoint; recovery did not differ based on substance use status.

**Discussion:**

SUD and especially substance dependence are common in psychotic disorders even in FEP patients. Evidence-based integrated care led to long-term improvement in patients with comorbid SUD and rate of recovery did not differ for patients with substance use.

## 1 Introduction

Schizophrenia-Spectrum-Disorders are associated with poor long-term outcomes as well as disability and severely affect the lives of patients and their families often from symptom onset, with almost half of all patients being diagnosed before the age of 25 years (Solmi et al., 2022). According to the theory of the “critical period”, which refers to the first 3–5 years of the illness (Birchwood et al., 1998), therapeutic efforts are thought to have the most pronounced effect during this phase, particularly when combined as part of a multi-component intervention and delivered in community-based settings (Secher et al., 2015; Correll et al., 2018; Frawley et al., 2023). Recovery from schizophrenia is one of the main goals in therapy, but still remains low, with just 20.8% of first-episode patients achieving clinical recovery in the long-term (Hansen et al., 2022). In the first episode of schizophrenia or in the early stages of the illness, patients generally respond significantly better to treatment (Takeuchi et al., 2018; Correll et al., 2022; Taipale et al., 2022) as the effects of many years of illness and functional decline are not yet that pronounced (De Winter et al., 2022). Nevertheless, even in the early phases, individuals with first episode psychosis (FEP) are a particularly vulnerable group for negative outcomes, including high rates of treatment disengagement, suicides, medication non-adherence, and comorbidities (e.g., substance use disorders; Doyle et al., 2014; Tiihonen et al., 2018; Rubio et al., 2021; Correll et al., 2022; Taipale et al., 2022; Yung et al., 2022).

Comorbid substance related disorders (SUD) have a particularly negative impact on outcomes in patients with FEP (Anderson et al., 2018; Hejberg et al., 2018; Simon et al., 2018) as demonstrated by higher rates of mortality and suicide, and SUD use is associated with non-adherence to treatment, which is one of the most important factors in having a relapse. Additionally, patients with comorbid SUD have a longer duration of untreated psychosis (DUP) and continued use in first-episode patients is associated with a plethora of negative outcomes including increased symptoms, adjustment difficulties, service treatment non-adherence, relapses, hospitalizations (Wisdom et al., 2011), and service disengagement (Horsfall et al., 2009; Kreyenbuhl et al., 2009; O'Brien et al., 2009; Conus et al., 2010). SUD can precede the onset of psychotic symptoms, increasing the risk of developing a psychotic disorder (Henquet et al., 2004; Manrique-Garcia et al., 2012; Tarricone et al., 2014; Wright et al., 2022), but SUD can also coincide with or start after the onset of the disorder (e.g., Burns, 2013).

Up to 70% of FEP patients suffer from comorbid SUDs (Rabinowitz et al., 1998; Verma et al., 2002; Lambert et al., 2005; Addington and Addington, 2007; Wisdom et al., 2011) and the most commonly used substances are cannabis (lifetime rates ranging between 22% and 47%, Mueser et al., 1990, 1992, 2000) and alcohol (about 30%, Wisdom et al., 2011; Oluwoye et al., 2019; Langlois et al., 2021). There is consistent evidence that using cannabis in FEP can lower the threshold for experiencing new relapses or hospitalizations (Tarricone et al., 2014; Patel et al., 2016; Schoeler et al., 2016a,b) and triggering psychotic symptoms (van Dijk et al., 2012). Regarding long-term outcomes, there is less consistent evidence on the effects of continuous, reduced or stopped substance use, with non-users having more pronounced negative symptoms than users (Peralta and Cuesta, 1992; Quattrone et al., 2021). There is also mixed evidence that cannabis use predicts worse psychosocial functioning in FEP (Wright et al., 2022).

Significant evidence exists regarding the efficacy of treatment for FEP. In a recent meta-analysis of ten randomized trials (*n* = 2,176 patients), early intervention services were associated with better outcomes than TAU at the end of the observation period regarding all studied outcomes (Correll et al., 2018). These services normally require multiprofessional teams offering multimodal treatment, comprising psychotherapeutic, psychosocial and pharmacological interventions aiming at reducing symptoms, improving functional outcomes, and thereby minimizing the effects of the disorder on long-term disability (Hyatt et al., 2022). Treatment should be offered in a coordinated and integrated way to prevent patients getting lost among the different health care providers and should also comprise treatment of comorbid SUDs (Ruppelt et al., 2020; Hyatt et al., 2022). There is also a large body of evidence on the efficacy of early intervention for FEP (Hyatt et al., 2022), which can be successfully implemented under real-world conditions (Posselt et al., 2021).

To optimize and coordinate multiprofessional treatment of patients with severe mental disorders, our study group developed the ACCESS model of integrated care. In our previous ACCESS I (1-year follow-up compared to standard care, Lambert et al., 2010) and ACCESS II studies (4-year follow-up with continuous Integrated Care, Schoettle et al., 2014; Lambert et al., 2015), it was shown that the ACCESS treatment model significantly reduced service disengagement, medication non-adherence, and involuntary admissions, and improved psychopathology, functioning, quality of life, and satisfaction with care, while being cost-effective compared to standard care (Lambert et al., 2015; Rohenkohl et al., 2022).

The ACCESS early detection and integrated care model for adolescents and young adults (ACCESS III) is based on the original ACCESS integrated care model for multiple-episode patients. Patients being treated with the ACCESS III model (Early Detection plus Integrated care, EDIC) had significantly higher rates of a combined symptomatic and functional remission compared to treatment in standard care and a better outcome after 1 year of treatment. Increased remission rates were predicted by being treated with integrated care (*OR* = 6.8, *p* < 0.001), while younger age predicted non-remission (*OR* = 1.1, *p* = 0.038). DUP was reduced and this reduction of DUP plus integrated care seemed to outweigh the negative influence of DUP on outcomes (Lambert et al., 2017).

Although no direct causal relationship to certain interventions can be drawn, the clinically meaningful effects in our study were probably related to the highly intensive and need-adapted integrated care interventions, conducted by the interdisciplinary therapeutic assertive community treatment (TACT) team with a focus on high-quality psychopharmacological and psychotherapeutic treatment. There is scarce information about treatment and differential outcomes of patients with dual diagnoses (psychotic and SUDs) in integrated treatment systems (Brunette and Mueser, 2006; Drake, 2008; Hunt et al., 2019; Abufarsakh et al., 2023; Wright et al., 2022). Penzenstadler et al. (2019) performed a systematic review of assertive community treatment (ACT) interventions for patients with SUD by analyzing randomized controlled studies. Although most of the patients had a severe mental disorder with SUD, the review did not focus on psychotic disorders and SUD. The results of the few RCTs were mixed; treatment engagement was higher for ACT in four studies and in two datasets, a superior effect on hospitalization rates was found.

In a recent systematic review on ACT in patients with severe mental illness (SMI) and SUD, although not directly comparable to our patient group, mixed results were found in most of the studies, which additionally were assessed to be of low quality (Abufarsakh et al., 2023). Nine out of 12 RCTs reported a decrease in substance use severity at follow-up, but no superior effect of ACT over comparison groups could be shown. Among eight cohort studies, only three demonstrated a significant decrease in alcohol severity or use (Abufarsakh et al., 2023).

In our ACCESS II study with mainly multi-episode patients, those with or without SUD improved both significantly over four years in all outcome parameters. However, patients with substance dependence showed significantly worse outcomes in psychopathology (*p* < 0.001), functioning (*p* = 0.006) and quality of life (*p* = 0.026). Regarding achieving recovery, comorbid substance use dependence was the only significant predictor for non-recovery (*OR* = 0.462, *p* = 0.048; Ruppelt et al., 2020).

In the ACCESS III study, presence of comorbid SUDs was explicitly not used as an exclusion criterion to increase the generalizability of results. As such, we were particularly interested in the course and outcome of patients in the early phase of psychosis with SUD compared with those individuals without SUD. Furthermore, we stratified patients according to their pattern of substance use, whether they reported (1) never using substances, (2) decreased or discontinued substance use or (3) ongoing substance use after 1 year of follow-up.

This article focuses on three main questions: (1) Are course of illness and course quality of life of patients in the early phase of a psychotic disorder with and without comorbid SUD in an evidence-based integrated care model comparable? (2) Do patients with differing patterns of substance use over the course of treatment (i.e., persistent, reduced/remitted or no substance) differ regarding course of illness and quality of life? (3) Do patients with comorbid SUD have differing rates of combined symptomatic and functional long-term recovery (after 1 year of treatment) as patients without SUD?

## 2 Methods

### 2.1 Study design and sample

The detailed study design and results of the ACCESS III study were published previously (Lambert et al., 2018). The ACCESS III study evaluated the extension of the original ACCESS integrated care model by implementation of a broad early detection initiative (e.g., a trialogue “awareness campaign”, implementation of a cross-age and interdisciplinary mobile early detection team) through close collaboration with child- and adolescent psychiatrists. It included severely ill adolescents and young adult patients with early psychosis in the age range of 12–29 years, living in the urban catchment area (e.g., including central station) of about 300,000 inhabitants of the university medical center Hamburg-Eppendorf (UKE). The ACCESS III study was a prospective, non-randomized, single center, 1-year cohort study comparing early detection plus integrated care (EDIC; conducted in the years 2011–2015) with a quasi-experimental historical control group having received standard care (SC; conducted in the years 2005–2008) (Table 1). The study was conducted as part of “psychenet—The Hamburg Network for Mental Health” (Härter et al., 2012), funded by the Federal Ministry of Education and Research, Germany (BMBF). The trial received ethics approval (Ethikkomission der Ärztekammer Hamburg, Approval number: PV3642) and was registered (ClinicalTrials.gov, Identifiers: NCT02037581, Protocol ID: 01KQ1002B; initial release: 1/14/2014; status: completed 7/13/2015).

**Table 1:** Description of the ACCESS-III-model of integrated care.

**Intervention**	**Intervention condition: Early Detection plus Integrated Care (EDIC)**
Characteristics	Hamburg Model for adolescents and young adults (12–29 years)
Participating hospitals	• University Medical Center Hamburg-Eppendorf (UKE)
Participating departments	UKE: • Department of Psychiatry and Psychotherapy (AP) • Department of Child- and Youth Psychiatry and Psychotherapy (CYP)
Participating institutions within the departments	Early detection service: • Early Detection Service for Mental Disorders (AP/CYP) Inpatient care: • Specialized psychosis inpatient unit (AP) • Acute care inpatient unit (AP) • Adolescent psychiatry inpatient unit (AP/CYP) • Youth inpatient unit (CYP) Day-clinic care: • Specialized psychosis day-clinic unit (AP/CYP) • Day-clinic for first-episode psychosis (AP/CYP) Outpatient care: • Specialized psychosis outpatient center (AP) • Acute care inpatient unit (AP) • Adolescent psychiatry inpatient unit (AP/CYP) • Youth inpatient unit (CYP) Outpatient network: • Private psychiatrists and psychotherapists (AP/CYP) • Psychosocial contact services • Youth help services • School-psychology services
Integrated Care including TACT	• Implementation of a cross-age and interdisciplinary Integrated Care model including a TACT team
**ACT team fidelity**	
Maximum Full-time Employee caseload	• 15–25
Staff fidelity and skills	• Consultant psychiatrists, psychiatrists, psychologists, nurses, social worker
Staff skills	• Diagnosis-specific training in pharmacotherapy, cognitive behavioral (CBT), dynamic, and/or family psychotherapy, pharmacotherapy
Work style	• Shared caseload, patients are discussed in daily team meetings, weekly internal and external supervision, regularly patient-centered network meetings
Availability	• Extended hours (8 a.m. to 6 p.m. Monday to Friday) and 24-h crisis telephone and 24-h emergency service within the Department
Contact with clients	• High frequent face-to-face contacts, assertive engagement, shared-decision making, “no drop-out” policy
Main interventions	• Case management, home treatment, individual, group and family psychotherapy, psychoeducation, pharmacotherapy, social work

AP, Adult Psychiatry; CYP, Child- and Youthpsychiatry; CBT, cognitive behavioral therapy.

In this article, we focused our analysis on the patients in the intervention group (EDIC, *N* = 120). In the historical control group insufficient information for analysis on substance use was available. Therefore, we did not go into further detail about the historical control group. Our results refer exclusively to the EDIC group. Those patients with a (history of) comorbid SUD (dependence and abuse) upon entry into the study were divided into three groups based on (persistence of) substance use over the course of treatment; (1) no substance use, (2) decreased or remitted substance use, and (3) persistent substance use at levels commensurate to those at the study entry.

### 2.2 Assessments and measurements

All patients who were willing to participate were informed about the study aims and procedures according to ethical principles and signed written informed consent. If the patient was under the age of 18, parents/guardians were also informed about the study and signed written informed consent (for further details see Lambert et al., 2017).

**Figure 1:**
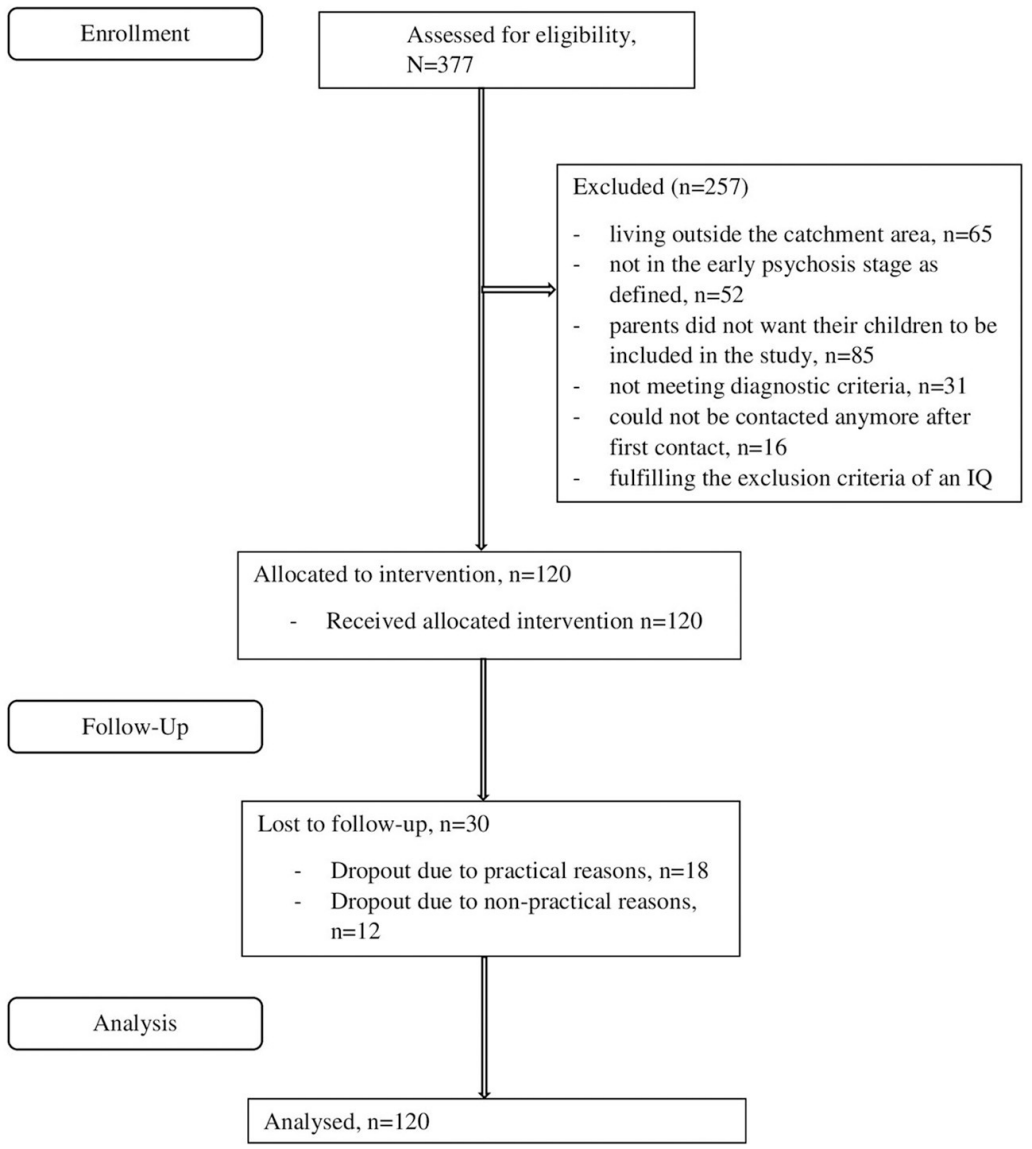
Flow chart.

Examination time points were screening, T0 (baseline), T1 (3 months), T2 (6 months), and T3 (12 months, study endpoint). The screening process included the evaluation of inclusion and exclusion criteria, patient information on the study content, and patients consent/assent for study participation (Figure 1). Data were collected by external raters.

Inclusion criteria included: (1) age 12–29 years, (2) sufficient knowledge of the German language, (3) early psychosis stage defined as a duration of ≤2 years between the first treatment with antipsychotics and study inclusion irrespective of adherence to treatment, (4) presence of a schizophrenia-spectrum-disorder according to DSM-IV-TR (Wittchen et al., 1997; First et al., 2002) or of affective disorders with psychotic symptoms; (5) written informed consent by the patient (≥18 years) or by guardians with written informed assent by the patient (12–17 years). Exclusion criteria included presence of one of the following diagnoses according to DSM-IV-TR (Wittchen et al., 1997; First et al., 2002): Alcohol- or substance-induced psychosis (concurrent alcohol or substance abuse or dependence were allowed), psychotic disorder due to a medical condition, pregnancy and mental disability.

In short (for further details please see Lambert et al., 2017, 2018), psychiatric diagnoses including all comorbid mental disorders, as well as SUDs, were assessed with the Structured Clinical Interview I and II for DSM-IV (Wittchen et al., 1997; First et al., 2002), demographic characteristics were assessed with the Early Psychosis File Questionnaire (EPFQ) (McGorry et al., 1990a; Lambert et al., 2005), DUP with the Royal Park Multidiagnostic Instrument for Psychosis Part I and II (McGorry et al., 1990a,b), and childhood adversities with an instrument adapted by Green et al. (2010). Somatic disorders at baseline, social support, and suicide attempt diagnoses at baseline were assessed using ICD-10-GM [German Institute of Medical Documentation and Information (DIMDI), 2016]. At T0, T1, T2, and T3 the following scales were assessed: psychopathology with the Positive and Negative Syndrome Scale (PANSS; Kay et al., 1987), functioning level with the Global Assessment of Functioning Scale [GAF; American Psychiatric Association (APA), 2000], severity of illness for schizophrenia spectrum disorders with the Clinical Global Impression Scale—Schizophrenia (CGI; Haro et al., 2003), quality of life with the Quality of Life Enjoyment and Satisfaction Questionnaire (Q-LES-Q-18; Ritsner et al., 2005), substance use with the European Addiction Severity Index (EuropASI; Scheurich et al., 2000).

### 2.3 Definition of remission

Combined symptomatic and functional long-term remission was met when the following conditions were fulfilled at T2 (6 months) and T3 (12 months):

1. Symptomatic remission of negative and positive symptoms of psychosis according to the criteria defined by Andreasen et al. (2005): PANSS items p1, p2, p3, n1, n4, n6, g5, and g9 each rated with a value of ≤ 3 points (no greater than “mild”) for ≥ 6 months.
2. Functional remission according to the criterion by Albert et al. (2011), measured with the GAF and fulfilled when a value of ≥ 60 points persisted for ≥ 6 months.

### 2.4 Change in SUD

Change in substance use was divided into the following three groups, according to the definition by Lambert et al. (2005). The quantity of all used substances was collected with the EuropASI (Scheurich et al., 2000).

i) No SUD (SUD-no) defined as no baseline SUD,
ii) Decreased or remitted SUD (SUD-rem) defined as a decrease in quantity of ≥50% or remission of baseline substance use at 12-month follow-up, and
iii) Persistent SUD (SUD-per) defined as increased SUD (≥50% increase in quantity and frequency of substances used); or unchanged SUD (<50% decrease or 50% increase from baseline substance use).

### 2.5 Statistical analyses

Descriptive analyses consisted of absolute and relative frequencies in categorical variables and either means and standard deviations (SDs) or medians with upper and lower quartile for continuous variables.

Baseline differences between groups (history of comorbid SUD vs. no history of comorbid SUD) were assessed using a *t*-test for independent samples when the dependent variable was continuous. Categorical variables were assessed with χ^2^-tests.

Changes over time in course of illness and course of quality of life were evaluated in mixed model repeated measures (MMRM), considering follow-up times (T1, T2, T3) as repeated measures, the patients as the random effect, the group (because of the explorative character of the analysis we separated comorbid SUD into substance abuse and dependence), change in SUD (SUD-no, SUD-rem, SUD-per) and time as fixed effects, and the baseline values (T0) of the dependent variable as covariate. Models were controlled for sex and age. Outcomes were changes from baseline in PANSS total and sub scores, CGI score, GAF and Q-LES-Q-18. The *time x group* interaction (comorbid dependence or abuse) as well as *time x change in SUD* interaction were examined. If the interaction was not significant, it was eliminated from the model using backward selection. Baseline values were used as covariates to minimize the variance. The Estimated Marginal Mean (*EMM*), Standard Error (*SE*), main effect (*F*), significance levels (*p*), and confidence intervals (*CI*) are reported.

Fisher's exact test was used to compare the differences in remission by group. Additionally, a binary logistic regression analysis was conducted to estimate the odds that the symptomatic and functional remission criteria were fulfilled at the study endpoint, using the following five predictor variables: (1) diagnosis (affective vs. non-affective psychosis), (2) age, (3) DUP, (4) substance dependence or substance abuse and (5) change in SUD. Further, we conducted a subgroup analysis of diagnostic groups. Results were represented using odds ratios (*OR*s) with 95% confidence intervals (*CI*s). A two-sided significance level at *p* < 0.05 was used to determine the association between the predictors and the primary outcome variable (sustained combined remission). Statistical analyses were performed with SPSS Version 27.0 (IBM Corp. Released, 2020).

## 3 Results

### 3.1 Sociodemographic and baseline characteristics

A total of 377 patients were screened for eligibility in the EDIC-group (Figure 1). Of these, 120 patients (31.8%) were included (for further details see Lambert et al., 2017); baseline descriptors of the patients including diagnoses are displayed in Table 2. Patients were on average 21 years old (*M* = 20.9, *SD* = 4.2) and approximately 20% were younger than 18 years.

**Table 2:** Baseline characteristics of all patients (*N* = 120) and in comparison without (*n* = 46) and without (*n* = 74) comorbid SUD.

**Demographic details**	**All patients (*N* = 120)**	**Without SUD (*n* = 46)**	**With SUD (*n* = 74)**	***p*-value**
**Demographic details**				
Age, years, mean (SD)	20.9 (4.2)	20.4 (4.9)	21.3 (3.8)	0.265
Age < 18 years, *n* (%)	26 (21.7)	16 (34.8)	10 (13.5)	**0.006**
Sex, male, *n* (%)	63 (52.5)	21 (45.7)	42 (56.8)	0.263
Married/in a relationship, *n* (%)	24 (20.0)	8 (17.4)	16 (21.6)	0.644
At work/in school, *n* (%)	73 (60.8	33 (71.1)	40 (45.9)	0.058
Years in school, mean (SD)	10.8 (2.0)	10.4 (2.2)	11.1 (1.8)	0.061
**Diagnostic details at baseline**				
Age of illness onset, mean (SD)	19.8 (4.2)	19.2 (5.0)	20.1 (3.7)	0.271
Duration of untreated psychosis, weeks (Median Quartiles)	16.4 (3.9; 53.2)	18.7 (5.0; 53.8)	13.4 (3.5; 53.2)	0.440
Inpatient at baseline, *n* (%)	60 (50.0)	19 (41.3)	41 (55.4)	0.188
Psychotic disorder, *n* (%)^a^				0.071
Schizophrenia	77 (64.2)	29 (63.0)	48 (64.9)	
Bipolar disorder, most recent manic or mixed, severe with psychotic symptoms	16 (13.3)	4 (8.7)	12 (16.2)	
Schizophreniform disorder	13 (10.8)	6 (13.0)	7 (9.5)	
Schizoaffective disorder	7 (5.8)	1 (2.2)	6 (8.1)	
Major depressive episode, single or recurrent, severe with psychotic symptoms	5 (4.2)	4 (8.7)	1 (1.4)	
Delusional disorder	2 (1.7)	2 (4.3)	0 (0)	
Comorbid mental disorders other than SUD, *n* (%)^a^	79 (65.8)	25 (54.3)	54 (73.0)	**0.036**
Somatic disorders at baseline, *n* (%)^b^	37 (30.8)	14 (30.4)	23 (31.1)	0.941
Diagnoses of social support (Z-diagnoses)^b^				
At least one Z-diagnosis, *n* (%)	113 (94.2)	42 (91.3)	71 (95.9)	0.292
Number of Z-diagnoses, mean (SD)	4.3 (2.6)	4.1 (2.6)	4.4 (2.6)	0.551
Hospitalized in the past, *n* (%)	101 (84.2)	39 (84.8)	62 (83.3)	0.884
Psychotropic treatment at baseline, *n* (%)				
Antipsychotics	97 (80.8)	37 (80.4)	60 (81.1)	0.930
Antidepressants	27 (22.5)	10 (21.7)	17 (23.0)	0.875
Mood stabilizer	12 (10.0)	4 (8.7)	8 (10.8)	0.707
Full adherence with last medication, *n* (%)	85 (70.8)	35 (76.1)	50 (67.7)	0.398
**Other illness details**				
Family history of mental disorders, *n* (%)^c^				
Any mental disorder	82 (68.3)	30 (65.2)	52 (70.3)	0.563
Psychotic disorder^d^	29 (24.2)	11 (23.9)	18 (24.3)	0.959
Suicide attempts in the past (X-diagnoses), *n* (%)^b^	25 (20.8)	9 (19.6)	16 (21.6)	0.241
Childhood adversities, at least one, *n* (%)^e^	68 (56.7)	24 (52.3)	44 (59.5)	0.434

*p*-values marked in bold, if significant (*p* < 0.05).

a Assessed with Structured Clinical Interview for DSM-IV (Wittchen et al., 1997; First et al., 2002).

b According to ICD-10-GM criteria [German Institute of Medical Documentation and Information (DIMDI), 2016].

c First-degree relatives.

d Including schizophrenia spectrum disorders, bipolar disorder severe with psychotic symptoms, major depressive episode severe with psychotic symptoms.

e According to childhood adversity criteria (Green et al., 2010).

Of the 120 patients (25%), 30 were lost to follow-up due to practical (*n* = 18, 60%) or non-practical reasons (*n* = 12, 40%). Service disengagement for practical reasons was considered for example if the patient moved out (*n* = 11, 36.7%) or changed his place of treatment outside of the catchment area (*n* = 7 patients). A dropout for non-practical reasons was, for example, if a patient repeatedly refused further treatment despite the need and several attempts at reengagement (e.g., phone calls to the patient and potentially home visits by the assertive community treatment team).

### 3.2 Type of comorbid substance use disorder and rates of persistent, reduced and remitted substance use

A history of SUDs was found in 74 (61.6%) patients. Mean age at the retrospective assessed beginning of the SUD was 17.2 years (*SD* = 4.0). Significantly fewer patients with a comorbid SUD were younger than 18 years (*p* = 0.006) and patients with a SUD had significantly more comorbid mental disorders other than SUD (*p* = 0.036).

The criteria for any substance abuse was fulfilled by 53 (44.2%) patients and for any substance dependence by 46 patients (38.0%). Thirty-four patients with comorbid SUDs reported using one substance (28.3%), 29 patients reported using two substances (24.2%) and 11 patients reported using more than two substances (9.2%). The most commonly used substances among those with SUD (*n*= 74) were cannabis (abuse: *n* = 23 patients (31.1%), dependence: *n* = 46 patients (62.2%) and alcohol (abuse: *n* = 23 patients (31.1%), dependence: *n* = 5 patients (6.8%). Combined use of alcohol and cannabis was reported by 23 patients (19.2%). Furthermore, three patients (4.1%) used sedatives or stimulants, two (2.7%) used cocaine, and one 1.3%) used hallucinogens. Additionally, 23 patients had another psychoactive substance related disorder, details are displayed in Table 3. Eighty patients used nicotine (nicotine use was not included into further analyses).

**Table 3:** Details of comorbid SUD (*n* = 74).

**SUD details**	**With SUD (*N* = 74)**
Number of comorbid SUD, mean (SD), range	1.7 (0.8), 1–4
Abuse, any, *n* (%)	53 (44.2)
Dependence any, *n* (%)	46 (62.2)
Age of start, mean (SD), range	17.2 (4.0), 10–28
**Substance abuse in detail**, ***n*** **(%)**	
Alcohol	23 (31.1)
Cannabis	23 (31.1)
Sedatives, Hypnotics	2 (2.7)
Cocaine	2 (2.7)
Stimulants	1 (1.4)
Hallucinogen	1 (1.4)
Other psychoactive substance	21 (28.4)
**Substance dependence in detail**, ***n*** **(%)**	
Alcohol	5 (6.8)
Cannabis	46 (62.2)
Sedatives, hypnotics	1 (1.4)
Stimulants	2 (2.7)
Other psychoactive substance	2 (2.7)

After one year of treatment, *n* = 25 (27.7%) patients reported persistent substance use (SUD-per), *n* = 31 (34.4%) reported decreased or remitted substance use (SUD-rem) and *n* = 34 (47.7%) reported no substance use at any point during or prior to the study (SUD-no).

### 3.3 Multifactorial course of patients with and without SUD after 1 year of treatment in EDIC and influence of persistent, reduced or remitted substance use

#### 3.3.1 Psychopathology, severity of illness, and functional status

During follow-up, significant improvements in psychopathology, severity of illness and functional status at one-year follow-up were found in all patients for the PANSS Total (*p* < 0.001), Positive (*p* = 0.001), Negative (*p* = 0.001), and Global (*p* < 0.001) rating, as well as the CGI total score (*p* < 0.001) and GAF (*p* < 0.001), when controlled for age and gender. There were also no significant differences with regard to these ratings between patients with a comorbid SUD (comorbid substance abuse or comorbid substance dependence) vs. those without. Furthermore, there were no significant differences between the three subgroups (SUD-per, SUD-rem, SUD-no) regarding the course of the PANSS ratings over the entire follow-up period (including all subscales), CGI total score or GAF.

#### 3.3.2 Quality of life

**Figure 2:**
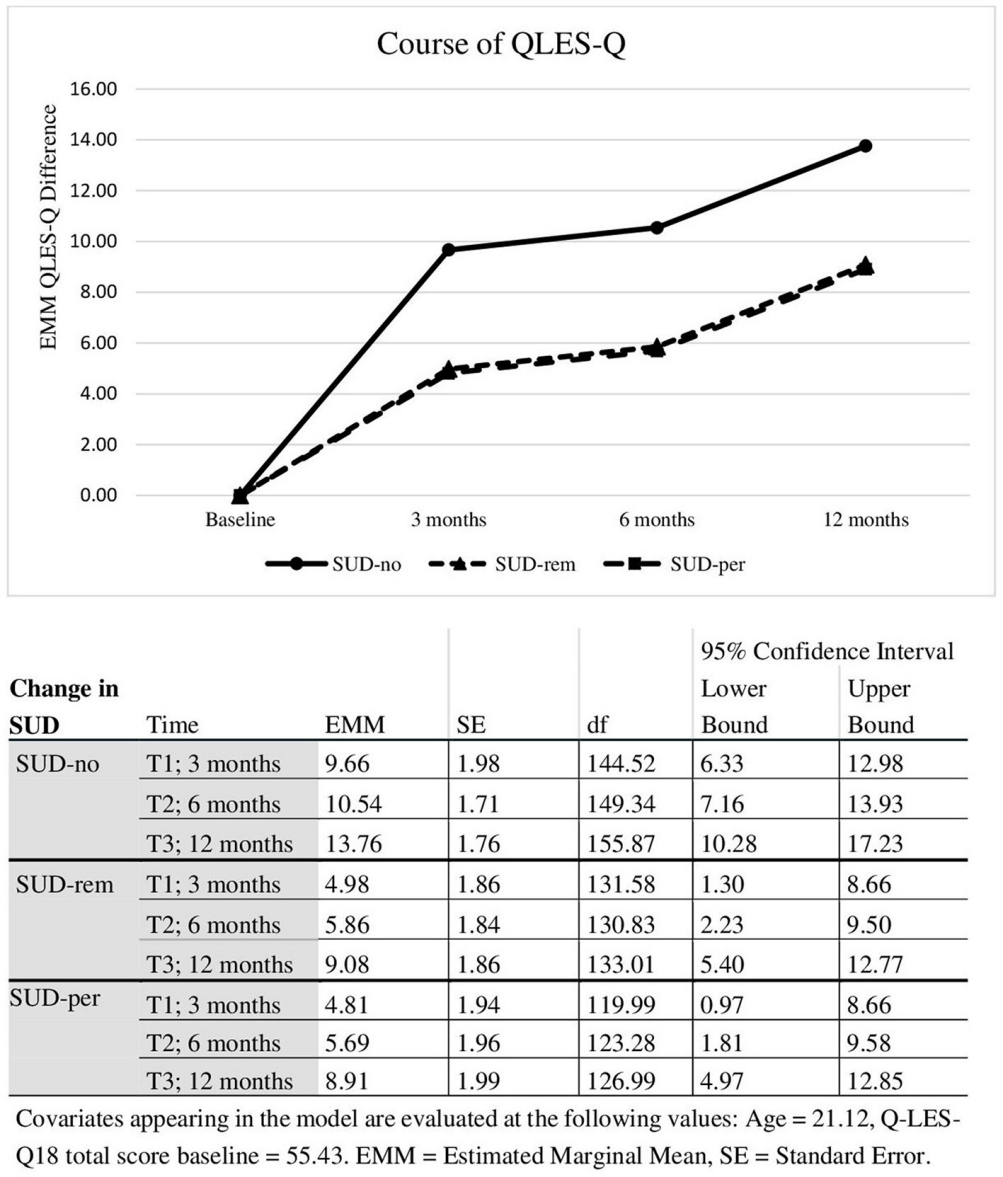
Course in quality of life differed by changed in SUD.

At follow-up, QLES-Q improved significantly at 1 year follow-up (*F* = 3.22, *p* = 0.044). Mixed models repeated measurements (Figure 2) indicated larger improvements (significant group effect of change in SUD, *F* = 3.10, *p* = 0.05) in the SUD-no group and compared to the group SUD-per (*p* = 0.038) and to the group SUD-rem (*p* = 0.036). There was no difference between the groups SUD-rem and SUD-per (*p* = 0.943). Neither substance dependence nor substance abuse had an influence and were thereby eliminated from the model.

### 3.4 Combined symptomatic and functional remission after 1 year

According to analyses with available cases, 48.9% (*n* = 44) of all patients in the EDIC group had achieved a combined symptomatic and functional remission for 6 months at the 1-year follow-up assessment. In the sensitivity analysis (LOCF), a combined symptomatic and functional remission for 6 consecutive months was achieved in 41.7% (*n* = 50) of the patients. Of those patients fulfilling the combined remission criteria, 30% (*n* = 15) of the patients had no SUD at baseline and 70% (*n* = 35) of the patients had a comorbid SUD (SUD-abuse: *n* = 24 patients; SUD-dependence: *n* = 21; SUD-no: *n* = 15, SUD-rem: *n* = 18, SUD-per: *n* = 14). There was no significant difference between patients with and without a comorbid SUD (neither dependence nor abuse). In the binary logistic regression (conducted using LOCF cases), neither a comorbid substance use dependence or abuse nor a change in substance use was found had a significant impact on the 6-months psycho-functional remission at the end of the follow-up period.

Overall, there were very low rehospitalization rates in the entire sample (*n* = 16). There was no significant difference in the rehospitalization rates between the patients without SUD (*n* = 6) compared to the patients with SUD (*n* = 10; substance abuse *n* = 3 and substance dependence *n* = 7).

## 4 Discussion

The ACCESS-III study assesses the effectiveness of the ACCESS integrated care model for FEP or patients in the early phase of a psychotic disorder. In the present analyses, we sought to study the course of illness in patients with and without comorbid SUD and, specifically, to determine whether decreased, remitted or persistent substance use had an impact on the rates of a combined symptomatic and functional long-term recovery compared with patients without SUD.

In our cohort, most patients were diagnosed with schizophrenia, about one-fifth were under the age of 18 and most patients were severely impaired with high levels of psychopathology and impaired functioning, no matter if they had a comorbid SUD or not, comparable to patient groups in other studies (Delespaul, 2013; Addington et al., 2015; Gühne et al., 2015). Among the total sample, more than 90% had a diagnosis of at least one psychiatric comorbidity, 62% had a comorbid SUD, 70% reported experiencing traumatic events in the past, and 80% had at least one chronic somatic comorbidity. Most (80%) were also functionally impaired and currently unemployed (Petersen et al., 2008; Fulford et al., 2013; Schlosser et al., 2014; Bond et al., 2015; Ajnakina et al., 2021). These rates of comorbid psychiatric and somatic disorders, as well as the (low) employment rates are comparable to real-world cohorts in other studies (Gates et al., 2015; Ajnakina et al., 2021).

Patients used on average two different substances and most of them fulfilled the criteria for substance dependence. Alcohol and cannabis were the most common used substances, which is in line with the international literature (Abdel-Baki et al., 2017; Oluwoye et al., 2019) and cannabis was the most common substance in our cohort in those with a substance dependence.

Around 24–74% of FEP patients have a lifetime SUD (e.g., Kovasznay et al., 1997; Rabinowitz et al., 1998; Kavanagh et al., 2004; Van Mastrigt et al., 2004; Lambert et al., 2005; Wade et al., 2005; Mauri et al., 2006; Addington and Addington, 2007; Barnett et al., 2007; Sara et al., 2013). Particularly, use of cannabis in FEP individuals is reported to be slightly higher and alcohol use slightly lower compared to multi-episode samples (Koskinen et al., 2010; Ruppelt et al., 2020), which is in line with our findings. Cannabis use is particularly problematic as cannabis increases the risk of developing a psychotic disorder (Di Forti et al., 2009, 2019) and users have a two to four time greater risk of developing psychotic disorders than non-users (Henquet et al., 2005; Moore et al., 2007; Marconi et al., 2016). The patients in our cohort started consuming substances on average at 17 years. Although no direct causal conclusions in our cohort can be drawn, it is well-known that particularly early cannabis consume is a risk factor for developing psychosis in patients with a clinical high risk for psychosis and that up to 25% of those with a substance-induced psychotic disorder will develop schizophrenia (Correll et al., 2022).

*(1) Are course of illness and course quality of life of patients in the early phase of a psychotic disorder with and without comorbid SUD in an evidence-based integrated care model comparable?*

We assessed whether comorbid SUD affected several mental health outcomes over the follow-up time of 1 year, including course of psychopathology, severity of illness, global functioning, and quality of life.

Follow-up ratings on the PANSS, CGI, and GAF indicated significantly and clinically improved psychopathology and functioning among all patients, as well as significantly improved quality of life. No group differences regarding course of illness between patients either with or without comorbid SUD or between patients with substance abuse or substance dependence were found.

Although conclusions regarding causal effects cannot be made due to the non-randomized and single group design of the study, over the follow-up time, treatment in EDIC seemed to be helpful for a wide range of (mostly) severely ill patients within the early phase of psychosis on several important outcome measures independent of SUD comorbidity status. This finding was surprising as, based on our previous study (Ruppelt et al., 2020), we expected those with a comorbid SUD, especially substance dependence, would benefit less from treatment than those patients without a comorbid SUD. Although not directly comparable because of different treatment settings and follow-up times, many studies have shown that long-term outcomes are generally worse in patients with comorbid SUD (Wisdom et al., 2011; Anderson et al., 2018; Hejberg et al., 2018; Simon et al., 2018). Substance dependence in patients with FEP is more commonly associated with the presence of positive vs. negative symptoms compared with non-users (Ringen et al., 2016; Seddon et al., 2016; Quattrone et al., 2021; Ricci et al., 2021a,b) and the course of illness is often poorer regarding response, adherence, relapse rates and functioning (Patel et al., 2016; Ringen et al., 2016; Seddon et al., 2016; Schoeler and Petros, 2017; Hasan et al., 2020). On the other hand, the prospective outcomes in individuals with dual diagnoses using cannabis, for example, are not reported in all studies as consistently worse compared with those without SUD. There is mixed evidence that cannabis worsens all symptoms and there are recent studies showing that negative symptoms are more severe than in non-cannabis users with FEP (Peralta and Cuesta, 1992; Quattrone et al., 2021). In our previous study, which included patients in all phases of psychotic disorders, patients with a substance dependence or abuse improved significantly more than those without regarding severity of illness (CGI-S) scores and these improvements were mainly due to improvements in negative and depressive symptoms (Ruppelt et al., 2020). These surprising findings in the EDIC group, although speculatively, might be explained by the early intervention and treatment of both, the psychotic disorder, as well as SUDs, we have no knowledge about the exact amount of each of the substances that were consumed (e.g., amount of THC), probably not having a SUD for a long time could also be associated with better treatment outcomes. This means that younger patients are not as long exposed to their SUD as older patients are. Therefore, the psychotherapy that begins very early with EDIC might also have an early positive effect on the course of the SUDs. The family interventions could also have contributed to this, as they are known to have very good effects in FEP patients (Bighelli et al., 2021).

Although we do not know which treatment modules (e. g. the highly intensive and need-adapted integrated care interventions mainly conducted by the interdisciplinary TACT Team with a focus on high-quality psychopharmacological and psychotherapeutic treatment) work especially well for those with a comorbid SUD, patients in the ACCESS treatment model demonstrated therapeutic benefit across a wide range of illness severity and comorbid disorders such as SUD.

*(2) Do patients with differing patterns of substance use over the course of treatment (i.e., persistent, reduced/remitted or no substance) differ regarding course of illness and quality of life?*

In our patient cohort of 120 patients, 25 (27.7%) patients reported continued use, 31 (34.4%) reported reduced or remitted use, and 34 (47.7%%) reported no use. We analyzed whether this has an impact on any of the assessed outcome criteria. Change in psychosis symptoms (as measured by the PANSS), illness severity (CGI total score) and functioning (GAF) were not significantly different between the three subgroups (SUD-per, SUD-rem, SUD-no). The only outcome parameter which was affected by SUD was quality of life, with larger improvement found in the SUD-no group compared to the group SUD-per (*p* = 0.038) and to the group SUD-rem (*p* = 0.036).

Regarding cannabis use, in the RAISE-ETP study (Wright et al., 2022), participants who used cannabis sporadically were more impaired than those who used cannabis consistently or those who did not use. In contrast to the RAISE-ETP study, we did not only focus on cannabis in this analysis. Abdel-Baki et al. (2017) found significantly more improvement in quality of life in patients who never had SUD or stopped using substances compared to persistent users. These differences may also be caused by different definitions of change in substance use.

Our results regarding quality of life might be explained by less coping strategies and knowledge in dealing with stress in patients who use substances in the present and also in their past. Maybe 1 year of treatment might not be enough to analyse, whether these findings remain after more years with psychotherapy, therefore more research with longer treatment and an extended observation period would be helpful. In future research, the influence of other comorbid mental disorders, such as trauma related disorders, might be interesting, as for example trauma is also related with higher substance use and impaired quality of life (Schäfer and Fisher, 2022).

*(3) Do patients with comorbid SUD have differing rates of combined symptomatic and functional long-term recovery (after 1 year of treatment) as patients without SUD?*

Nearly half of the patients fulfilled the criteria for psycho-functional remission for the last 6 months at the 1-year follow-up, regardless of whether they had a comorbid SUD or change in substance use.

In a recent systematic review, a recovery rate of 20.8% was found among those patients with a first episode of schizophrenia (Hansen et al., 2022). The follow-up time was in the mean 9.5 years in this study and therefore significantly longer than in our study, but in the meta-regression none of the study characteristics could uncover the diverse reported recovery rates; age (*p* = 0.84) or year of inclusion (*p* = 0.93), follow-up time (*p* = 0.99), drop-out rate (*p* = 0.07), or strictness of the recovery criteria (*p* = 0.35, Hansen et al., 2022).

In contrast to our findings from the ACCESS II study regarding recovery (Ruppelt et al., 2020), there does not appear to be any impact of comorbid SUD on remission in these young first-episode patients. This could be due to the fact, that patients under the age of 18 were included into this study, and treatment (including psychotherapy, family interventions and 24/7 availability) was able to take effect much earlier, relatives were included very often and institutions for minors (like school psychologists) were integrated into the network.

### 4.1 Limitations

This is a single-center unblinded study and due to the lack of a control group, no causal conclusions can be drawn. Thus, the data must be interpreted as observational. Therefore, the raters were not blinded and there is no control group. We used external raters to assure assessment quality and to reduce—but not to fully avoid—social desirability bias and thus too positive ratings of psychopathology.

Data on substance use was based on patient self-report, which might be effected by social desirability. However, self-report of substance use has been shown to correlate well with objective measures (O'Farrell et al., 2003). A confounding factor could be the quantity of used substances before entering the treatment model; for example, we did not know the amount of THC in the cannabis having been consumed. SUD diagnosis was not re-evaluated at study endpoint, so newly developed SUDs were not detected. We did not know anything about the patients that discontinued the treatment in EDIC. The study was not primarily designed to assess the effect of substance use on various outcomes. One year of treatment might not be long enough to show the long-term effects of substance use, especially when it comes to recovery. It could also be that substance use varied over the course of the study; however, this was not captured in our analyses. Finally, the representativeness of the sample may be limited by the exclusion of homeless patients, who were, by definition of the catchment area, treated elsewhere.

### 4.2 Clinical implications

In this article, we sought to examine the impacts of comorbid SUD and change in substance use on long-term multidimensional outcomes including remission and recovery in patients with psychotic disorders treated for 1 year in the EDIC model. This secondary subgroup analysis of our ACCESS III cohort indicated that benefits of EDIC treatment was not affected by SUD status at baseline or substance use.

We can only hypothesize about which factors may explain the beneficial results; however, the nature of the treatment likely contributed to the positive outcomes. For example, patients are offered treatment with a TACT-team specially trained in psychosis treatment and embedded in integrated care offering a broad spectrum of treatment options for psychosis and comorbidities, which are administered in a need-adapted manner. Due to the high intensity of treatment with several outpatient contacts per week, it is possible to build a strong therapeutic relationship. The treatment team is committed to psychotherapy and family involvement and works in a recovery-oriented manner with the severely ill patients.

In summary, the results of our study among patients in early phase of psychosis are promising, but to draw causal conclusions, stronger evidence including a long-term RCT focusing on dual-diagnosis patients would be required. Such treatment models should focus more on additional treatment options for patients with SUD, such as having a SUD expert in the multiprofessional team.

## Data availability statement

The datasets used and/or analyzed during the current study are available on reasonable request. Requests to access the datasets should be directed to ML, lambert@uke.de.

## Ethics statement

The studies involving humans were approved by Ethikkomission der Ärztekammer Hamburg, ethik@aekhh.de, Approval Number: PV3642. The studies were conducted in accordance with the local legislation and institutional requirements. Written informed consent for participation in this study was provided by the participants or if necessary their legal guardians/next of kin.

## Author contributions

FR and DS drafted this article. FR conducted the statistical analyses under the advice of AD. ML and AK were leader of the ACCESS III project embedded in psychenet, therefore they designed the study. Process of data collection was supported by AD. DS and DL ensured the treatment of patients within ACCESS III. ML, AK, AR, VK, BS, JG, and GL revised the manuscript. All authors contributed to and have approved the final manuscript.
